# Modulating the surface and mechanical properties of textile by oil-in-water emulsion design

**DOI:** 10.1039/d1ra07961a

**Published:** 2022-01-13

**Authors:** Evangelia Argentou, Carlos Amador, Anju Deepali Massey Brooker, Serafim Bakalis, Peter J. Fryer, Zhenyu Jason Zhang

**Affiliations:** School of Chemical Engineering, University of Birmingham Edgbaston Birmingham U.K. B15 2TT UK z.j.zhang@bham.ac.uk; Procter & Gamble, Newcastle Innovation Centre Newcastle-upon-Tyne U.K. NE12 9TS UK; Department of Food Science, University of Copenhagen Rolighedsvej 26 Frederiksberg DK-1958 Denmark

## Abstract

The synergistic effect of oil viscosity and oil droplet size on the deposition profile of oil on cotton fabric was studied using polydimethylsiloxane (PDMS) as a model oil-in-water emulsion system. Under the same preparation conditions, low viscosity PDMS produced emulsions containing small droplets, which resulted in a uniform surface deposition profile, whilst high viscosity PDMS resulted in a localised deposition profile. Interfacial phenomena such as wicking and penetration of PDMS into cotton fabrics were found to be viscosity-dependent, which agrees with the surface deposition data. Both mechanical characterisation (friction, compression, stiffness) and consumer evaluation confirm that the fabrics treated by the emulsion containing low viscosity PDMS were preferred, suggesting that a homogeneous surface deposition and an excellent penetration profile of PDMS are critical for maximising tactile sensorial benefits, which could be accomplished by optimising the emulsion formulation to contain oil of low viscosity and small PDMS droplets.

## Abstract 2

The synergistic effect of oil viscosity and oil droplet size on the deposition profile of oil on cotton fabric was studied using polydimethylsiloxane (PDMS) as a model oil-in-water emulsion system.

## Graphical abstract

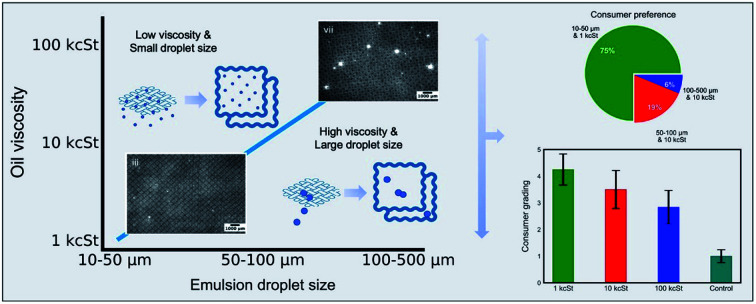

## Introduction

Actives of selected molecular architecture and molar mass are commonly used to modulate the surface and tribological characteristics of materials such as textiles, resulting in an array of benefits (*e.g.* softness, lubricity) that are desired by consumers.^1–6^ To enhance product sustainability without compromising product performance, it is critical to establish a comprehensive framework correlating formulation design to comfortableness perceived by the consumer.^7^ The physico-chemical transformations involved in the application of softener actives include surface deposition in liquid, rearrangement during drying, and controlled surface interactions in an ambient environment, all of which are underpinned by the surface and interface phenomena.

The ability to deliver and deposit actives on surfaces, alongside the knowledge that underpins the sensorial benefits of fabrics, are critical for the design of formulated products. For example, correlation between fabric friction and subjectively perceived touch properties was found for knitted, but not for woven fabrics, in a previous work.^8^ In the same study, relevant properties correlating friction and touch properties were identified as bending, thickness, and compressibility. Many theories have been proposed around the softening mechanism of actives such as polymers, and the most prominent one assumes the formation of a lubricating layer on the fibers upon deposition.^9–15^ Others have proposed that the softening benefit is due to the reduction in hydrogen bonding between water molecules and cotton fibres as a result of the deposition.^16–18^ Deposition of softening actives has also been explored extensively: some studies^19^ suggest that hydrophobic interactions due to the presence of long alkyl chains in cationic surfactants are the primary driving force for adsorption, whilst others^20^ have proposed that deposition is driven by electrostatic interactions. Attempts were made to correlate fabric tribology with smoothness and softness^21^ and others have put emphasis on studying the friction between human skin and fabrics.^22^ Polymers are often used in commercial fabric softeners since they can lead to improved textile properties such as wrinkle recovery, crease resistance, improved softness and better wear comfort without the disadvantages that accompany conventional softener types such as yellowing. Non-charged silicones with hydrophobic properties, such as polydimethylsiloxane (PDMS), are used extensively in the laundry industry.^6,23^

Surface deposition strategies of silicone have been studied extensively for the past three decades due to its ability to/cover the surface of the fibre (natural and synthetic ones), and consequently provide excellent softening properties, enhancing the sensory characteristics of fabrics.^9,23,24^ Deposition of PDMS on fabrics can occur in various ways, the most common being emulsions that contain PDMS/surfactant droplets. Surface deposition of actives is driven by complex physical and chemical phenomena during a wash process that are not well understood. The aqueous environment, the presence of ions, the porosity and roughness of the fabrics, the presence of surfactant monomers, the presence of shear forces as well as the hydrophobic nature of PDMS can all affect deposition.^24–26^

The impact of oil viscosity and emulsifiers on the resulting emulsion was investigated as early as 1990.^4^ It was reported that increasing oil viscosity could help to increase both the rates and extent of silicone deposition on to human hair, which was attributed to the increased attractive droplet–fibre interaction and ability to cover a large fibre surface area upon deposition.^27^ In a recent study, emulsions of organomodified silicones (OMS) were prepared to evaluate the effects of droplet size and charges.^27^ Panel tests suggested that the conventional emulsion (droplet size in the μm range) was favoured to microemulsions in which droplets were of nm size, which was attributed to the predominant surface deposition of silicone using the large emulsion. As a contrast, nano-emulsion of silicone was found to outperform micro- and macro-emulsions in terms of fabric softness, smoothness, cooler feel and thinness.^28^ Lastly, micro- and macro-emulsions of silicone were deposited on polyester fibres by Parvinzadeh and Hajiraissi,^2^ which suggested that macroemulsion was able to improve crease recovery due to the formation of silicone film that lubricates fibre–fibre friction.^1^ In the same study, microemulsion was found more effective in introducing crease resistance.

Upon deposition, migration of the oil droplets into fabrics is determined by wetting and wicking, both of which are multiscale phenomena that are determined by (i) the nature of the fibres such as surface charge, porosity, and hydrophobicity; (ii) characteristics of the fabrics, *e.g.* woven pattern and density; and (iii) surrounding environment such as pH, ionic strength. Electrokinetic properties of natural fibres have been studied extensively using techniques such as streaming potential, which has a significant impact on the deposition kinetics and characteristics of colloids.^27,29,30^ This equally is controlled by the oil profiles such that low viscosity and high interfacial energy would facilitate spreading.

Finally, the mechanistic relationship between amount, location, and characteristics of the deposited silicone and the sensorial benefits of fabrics remains unclear. This is partly due to the complex nature of skin tribology that is often correlated to a broad range of physical parameters. The sensory characteristics of the fabric are one of the components that determine the final product quality perception. The most important characteristics of textiles that seem to be correlated with performance attributes are appearance and “fabric handle”.^31,32^ The latter is defined as the perceived overall aesthetic quality that reflects the mechanical and physical properties of the fabric.^33^ It is commonly believed that there is a strong attraction between silicone and fabric, which enables silicone to spread and form a lubrication layer that alters the fabric tribology and therefore the “fabric handle”.^34–36^ It is therefore critical to consider the silicone distribution profile and its location on the fabric: should it be kept on the fabric external surface to intensify the skin-fabric lubrication or delivered into the fabrics for a homogeneous distribution.^29^

To design emulsion technology that contains either silicone or other alternative oils, it is therefore essential to establish a holistic approach that links oil characteristics with final sensorial perception, with a thorough consideration of the physical/chemical transformations involved. We therefore chose non-ionic silicone as a representative system for investigation in the present work. A series of emulsions of different oil droplet sizes were prepared as a function of silicone viscosity. The deposition profile of polydimethylsiloxane (PDMS) on fabrics was established using a fluorescent microscopy technique,^37,38^ complemented by elementary analysis and gravimetric measurements. Alteration to the physical properties of the fabrics was quantitatively investigated by mechanical testing and consumer evaluation.

## Experimental

### Materials

#### Polydimethylsiloxane (PDMS) emulsions

PDMS formulations containing 1 wt% polysorbate 80 (VWR, Leicestershire, UK), a non-ionic surfactant, and PDMS (Dow Corning) were prepared using a high speed mixer (Silverson L4RT, Silverson Machines Ltd., Chesham, UK) for 15 min at 3000 rpm. PDMS of various viscosity (10^3^, 10^4^, and 10^5^ cSt), of which density is 0.970, 0.974, and 0.977 respectively, was used. Both high (0.3 wt%) and low (0.03 wt%) PDMS concentrations were used to produced PDMS droplets of different sizes, replicating a simple fabric care delivery system.^39^ Fluorescent agent Pyrromethene 546 (PM546),^40–42^ pre-dissolved in acetone (0.05 wt%), was added in the PDMS at a concentration of 0.5 wt% for the deposition study. All emulsions were prepared using distilled water.

#### Fabrics

Cotton fabric WFK 10A (WFK Testgewebe GmbH, Krefeld, Germany) was washed in deionized water at 90 °C for three times, of which 8 cm × 8 cm square pieces were collected for small-scale wash and following characterisation. To assess sensorial characteristics, terry towels (33 cm × 33 cm Standard Textile Piano Key, Standard Textile, Ohio, USA) were selected for consumer response studies. The terry fabrics were washed three times with deionized water at 90 °C to remove any impurities.

### Methods

#### Small-scale wash deposition

To replicate a small-scale washing process,^43^ the prepared PDMS emulsions (800 g) were added into a tergotometer (Copley Scientific, Nottingham, UK), along with 15 g of 100% cotton fabric WFK 10A (WFK Testgewebe GmbH, Krefeld, Germany), for 10 min at 2 °C and 100 rpm stirrer speed. The fabrics were subsequently collected and left to dry overnight for further analysis.

#### Scanning electron microscopy

An environmental SEM (Philips XL-30 FEG) with Oxford Inca EDS, coupled with a Polar Prep 2000 cryo-stage, was used to investigate the presence of lamellar structural characteristics within the prepared silicone loaded capsules (SLCs).

Surface morphology and elementary analysis were carried out using a tabletop SEM (Hitachi TM3030Plus), that is equipped with both Secondary Electrons (SE) and Backscattered Electron (BSE) detectors, with 5 kV and 15 kV accelerating voltage.

#### Particle size characterisation

A particle size analyser (Mastersizer 2000, Malvern Instruments, Malvern, UK) was used to quantitatively evaluate the sizes of the dispersed SLCs emulsions, and the silicone droplets in the model formulations. Refractive Index value of 1.4 (ref. 44 and 45) was used for quantifying PDMS droplets in the emulsions. DECON working solution (5% w/v), followed by copious amount of deionized water were used to clean the instruments (tubing and measurement chamber) between measurements. For each condition, three repeats were conducted to obtain a representative distribution.

#### Fluorescence microscopy

A fluorescence microscopy methodology to evaluate the presence and distribution of surface deposition was developed from a previous study.^38,46^ PM546 is a lipophilic water insoluble dye that exhibits absorption and emission maxima at ∼495 nm and ∼505 nm respectively.^40–42^ The deposition profiles were assessed on dry fabrics 8–12 h after the treatment with the emulsions to mimic the line drying process and obtain consumer relevant deposition profiles. The data were collected with a Leica MacroFluo Z16 AP0 microscope (Leica Microsystems, Germany).

#### Contact angle measurements

A microscopic camera was used to evaluate the spreadability of 0.5 g of PDMS variants (10^3^, 10^4^, and 10^5^ cSt), deposited on the fabric by a fine pipette, as a function of the contact time. Images were acquired at 0, 30, and 60 seconds, and processed using the angle measurement function of the ImageJ software. Each PDMS sample was measured for at least 3 times.

#### Physical properties of fabrics

Friction, compression, and stiffness measurements that underpin the sensorial benefits of fabrics were conducted on the terry cotton samples (Standard Textile, Ohio, USA) that were washed in 1 L PDMS emulsions (0.3 wt%) for 1 h at 30 ± 1 °C. Five replicates were assessed for each condition.

Coefficients of friction (COF, *μ*_s_) were measured on rectangular (12 cm × 6 cm) fabric samples using a COF/Peel Tester (Thwing-Albert FP-2260). A 200 g spring sled (02250–4400), with additional load of 500 g, equivalent to 6.86 N applied load, with a geometry of 2 1/2′′ × 2 1/2′′ (63.5 mm × 63.5 mm), resulting in a contact pressure of 1701 Pa, was used to assess the COF for each fabric sample. The sliding velocity that was used was 0.8 cm s^−1^.

Compression measurements were conducted by measuring the force needed to compress a circular (10 cm diameter) fabric samples using an Electromechanical Testing System (MTS Insight 10). The traction capacity was at 10 kN and the speed used was 200 mm min^−1^. The sampling frequency was 100 Hz and the software Testworks was used to operate the machine and obtain the results. For that purpose, a round piece of the terry cotton (12 cm diameter and 5.5 mm thickness) was used.

A square piece (5 cm × 5 cm) of the fabric sample was used for stiffness measurements in ‘Taber’ stiffness units (g cm) with an industry standard 150-E Stiffness Tester (Taber Industries, New York, USA).^47^

#### Consumer evaluation

For the purposes of the sensory evaluation, terry cotton (Standard Textile, Ohio, USA) fabric swatches (20 cm *×* 20 cm) were washed with 1 L PDMS emulsions (0.3 wt%) for 1 h at 30 °C. Different PDMS emulsions were prepared for each viscosity variant (10^3^, 10^4^, and 10^5^ cSt). Prior to the sensory evaluation session, all the samples were dried under standard ambient conditions. Each individual evaluation session was performed under the same conditions. A panel of 16 untrained individuals age between 18–35 was selected. The assessment technique used for this study was a “blind handling”, with the panellists assessing the samples in random order. Each item was graded in terms of overall sensorial feeling on a linear scale structured from 0 to 5, and anchored with polar attributes at the ends of 1–5 (5: best overall feel, 1: worst overall feel). Each panellist was also asked to choose one fabric as their overall preference.

## Results & discussion

### Size distribution of the emulsified PDMS

**Fig. 1:**
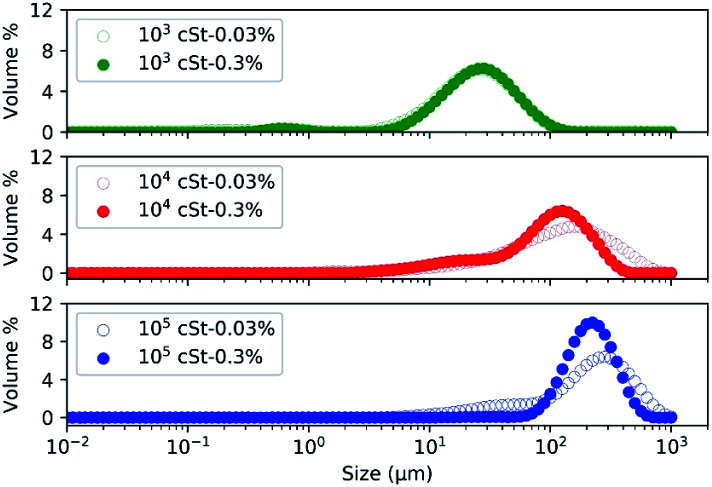
Size distribution of emulsified droplets using PDMS of different viscosity: (i) 10^3^, (ii) 10^4^, (iii) and 10^5^ cSt at two concentrations: 0.03% and 0.3%.

Previous studies^26,48–51^ suggest that the size of the emulsified PDMS droplets is determined by the amount of emulsifier, the emulsification time, the shear force applied, the amount and the viscosity of PDMS, of which the last two parameters were considered in the present work. Corresponding size distribution profiles of the emulsified PDMS droplets, measured by a particle size analyser, are presented in Fig. 1.

The size profiles presented in Fig. 1 confirm that the viscosity of PMDS has a significant impact on the size distribution of the resulting droplets: emulsions prepared with PDMS of low viscosity (10^3^ cSt) contained smaller droplets in comparison to those prepared with PDMS of high viscosity (10^4^, and 10^5^ cSt). The median droplet sizes are 20–50 μm, 50–100 μm, and 100–500 μm from emulsions prepared by 10^3^, 10^4^, and 10^5^ cSt PDMS, respectively. The use of high viscosity PDMS led to the formation of large droplets due to the high molecular weight of the PDMS molecule. Since the emulsification process, including the amount of emulsifier, shear force and time, was kept consistent, the notable variation in the size profile is likely because PDMS of high molar mass would require more energy, more time, or higher levels of surfactant molecules to emulsify and generate small droplets, as observed in a previous study.^4,26^ The effect of PDMS viscosity on the size distribution of emulsion would have significant implication on the industrial process whereby energy, time, and resources need to be considered.

Concentration of the emulsified droplets can be estimated from eqn (1) below1
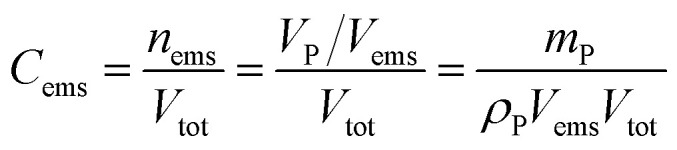
where *n*_ems_ is the number of PDMS droplets, *V*_tot_ is the total volume of the final emulsion, *V*_P_ is the volume of PDMS added initially, *V*_ems_ is the volume of a single emulsion droplet, *m*_p_ and *ρ*_P_is the weight and density of silicone oil added respectively. Assuming that all the emulsified droplets have the same spherical shape and radius, the volume of a droplet was calculated2
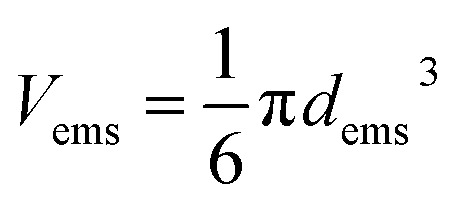


The diffusion coefficient of an individual emulsion droplet (*D*_ems_) can be estimated by the Stokes–Einstein equation,3
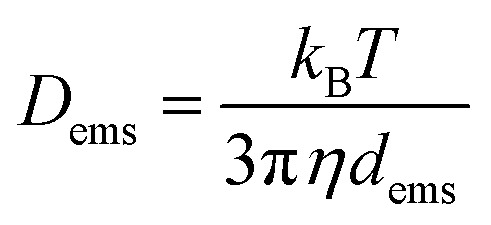


As for the small scale deposition, the fluid mechanics in the tergotometer was controlled under the same conditions. As such, the probability of collision (*f*_ems_) between PDMS droplets and fabrics can be estimated using the following equation,^52^ based on the frequency of impacts of diffusive flux on a finite disc electrode:4
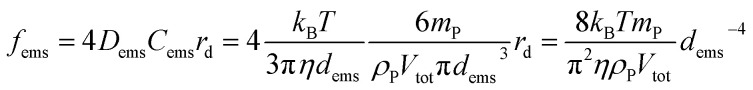
where *r*_d_ is the radius on a fabric disc. We recognise that such estimation was made based on a steady state condition, and a time-dependent factor could enhance the accuracy.^53^ However, the most important message here is that the collision frequency is significantly reliant on the size of the emulsified PDMS: the probability for a piece of fabric to contact an emulsified PDMS droplet of 200 μm is 10^4^ less than that containing droplets of 20 μm, or 256 times less than that of 50 μm.

#### Deposition profile of the emulsions on fabrics

Instead of considering the deposition as a macroscopic wicking and wetting process, we view the fabric deposition of emulsified PDMS in a washing process as a droplet impact and penetration phenomena, which consider the following parameters: size and viscosity of the droplets, porosity of the matrix, and the interfacial energy of the droplet/fibre.^54^ Both physical entrapment and interfacial spreading play important roles here.

• Increasing droplet size would result in deposition on the surface of the fabrics due to physical entrapment, which is equally determined by the porosity of the fabrics, whilst droplets of reduced size (less than the gap between yarns, 10–40 μm) are capable of penetrating into the fabrics.

• Interfacial energy of the droplet and fibres plays a critical role. This needs to be considered under the appropriate conditions. The surface energy of PDMS is consistent, as reported previously.^55^

• Lastly, viscosity of the oil influences the spreading of the PDMS droplets upon contact with the fabrics.

Recent study^54^ suggests a quantitative correlation between droplet spreading ratio, impact velocity, and fluid properties by interpolating between the capillary regime (∝ *W*_e_^1/2^) and viscous regime (∝ *R*_e_^1/5^) using a first order Padé approximation. This was further adjusted to account for low impact velocity droplet spreading, which is appropriate for the present work:5
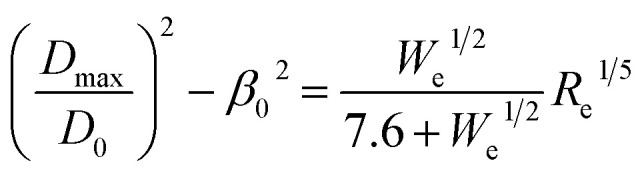
where *D*_ma*x*_ is the maximum spreading area, *D*_0_ is the initial size of the droplet, *β*_0_ is the value of the maximum spreading ratio at zero impact velocity, which is dependent on the liquid surface tension and surface wettability, *W*_e_ is Weber number (*W*_e_ = *ρD*_0_*v*^2^/*σ*), *R*_e_ is Reynolds number (*R*_e_ = *ρD*_0_*v*/*η*) for droplets of density *ρ*, shear rate viscosity *η*, and surface tension *σ*. The impact velocity is sufficiently low for the present work, and hence the maximum spreading (*D*_ma*x*_) is primarily determined by the Weber number (*D*_ma*x*_/*D*_0_ ∝ *W*_e_^1/2^). Given that the interfacial energy of silicone and water is consistent, we assume that its interfacial energy against the cotton fabrics used in the present work is similar, which results in *D*_ma*x*_ ∝ *D*_0_^3/2^. This implies that the maximum spreading diameter of droplet 200 μm could be approximately 32 times greater than that of a 20 μm droplet on the top surface of the fabric, which counters the much less collision probability estimated earlier.

**Fig. 2:**
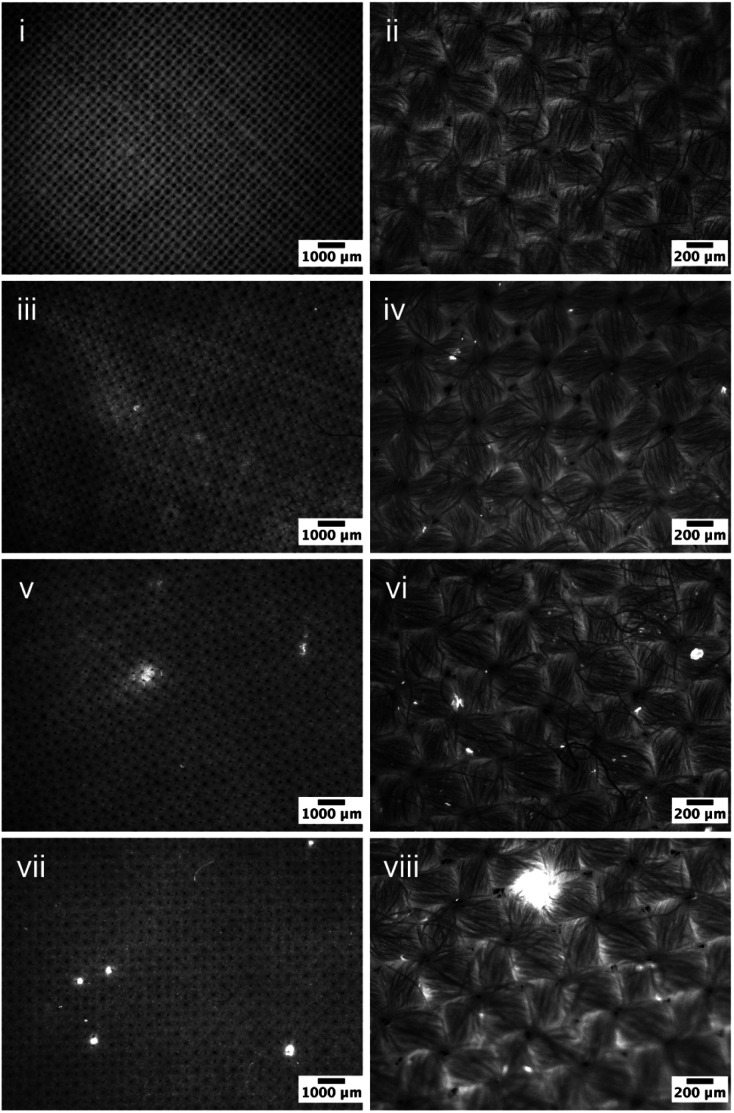
Fluorescent micrographs of (i–ii) blank WFK10A cotton, and the cotton samples exposed to 0.03% PDMS emulsion prepared using (iii–iv) 10^3^ cSt PDMS; (v–vi) 10^4^ cSt PDMS, and (vii–viii) 10^5^ cSt PDMS. The bright regions correspond to the presence of the surface deposited PDMS.

To verify the estimation above, fluorescence microscopy was used to evaluate the deposition profiles of the prepared PDMS emulsions on cotton fabrics following the small scale washing process and dried overnight (Fig. 2). Images acquired at two different magnifications are included (scale bars are 1000 and 200 μm respectively). The control sample is a standard plain weave cotton fabric that showed barely any fluorescence signal. Upon exposure to 0.03% PDMS emulsions using 10^3^, 10^4^, and 10^5^ cSt PDMS, bright regions were observed on the fabrics, which indicates the presence of deposited PDMS on the fabric surfaces since there was no other fluorescence agents introduced. The fluorophore used in the present work, Pyrromethene 546, is hydrophobic and binds strongly to PDMS, and hence can provide a solid evidence concerning the location of the deposited PDMS.

Very few bright regions were observed on the samples treated by emulsions containing 10^3^ cSt PDMS (Fig. 2iii and 2iv), indicating a limited presence of PDMS on the surface, which appears to be counter-intuitive as the probability of collision is much greater for the 10^3^ cSt PMDS droplets that those of 10^4^ cSt. It is very likely that the size of PDMS emulsions were significantly smaller than the size of the yarns (gaps between cotton fibre bundles), and hence were able to penetrate through the fabric into deeper regions. The presence of PDMS appeared to enhance as the viscosity of PDMS increased to 10^4^ cSt – not only there are large patches on the fabric surface (Fig. 2v), but also significantly increased numbers of bright spots of which the size is in the region of 20–60 μm, which are the results of PDMS deposition. As for the samples exposed to the formulation prepared using 10^5^ cSt, the number of large regions of deposition, several hundred microns in diameter, is notable (Fig. 2vii and viii). This change in the maximum spreading diameters is consistent qualitatively with the prediction based on eqn (5).

**Fig. 3:**
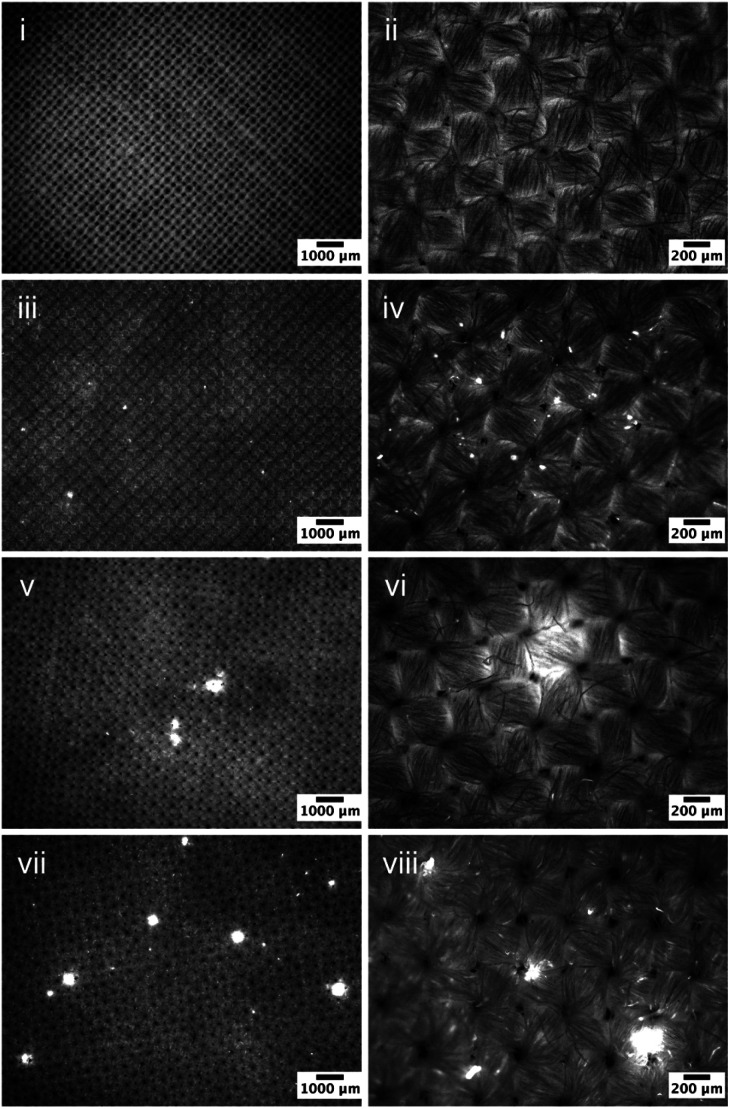
Fluorescent micrographs of (i–ii) blank WFK10A cotton, and the cotton samples exposed to 0.3% PDMS emulsion prepared using (iii–iv) 10^3^ cSt PDMS; (v–vi) 10^4^ cSt PDMS, and (vii–viii) 10^5^ cSt PDMS. The bright regions correspond to the presence of the surface deposited PDMS.

Fluorescence micrographs of the cotton samples exposed to 0.3%wt PDMS emulsions were subsequently acquired and are presented in Fig. 3. Very similar correlation can be found here: increased viscosity appears to result in an enhanced surface presence. It is worth noting that the increased concentration has a significant impact on deposition, for all formulations used, which is governed by eqn (4) whereby the collision frequency is proportional to the weight of the added PDMS, whilst the diameter of the emulsified droplets remains the same. Formulations prepared with 10 kcSt PDMS exhibited an increasingly uneven deposition profile on the fabric with the presence of large PDMS rich regions between 50-200 μm (Fig. 3v, vi). Such heterogeneity became more severe on fabrics treated by formulations using 100 kcSt PDMS: large PDMS being entrapped between the yarn intersections or deposited at the yarn surface (Fig. 3vii, viii). Their diameters are between 100-500 μm. The Fig. 3viii depicts the area occupied by the droplets of sizes 50–200 μm. Areas occupied by larger droplets can be seen in Fig. 3vii and could not be captured in higher magnification in high quality.

Observations on fabrics exposed to PDMS emulsions prepared can confirm the effects of PDMS viscosity on the surface deposition, on both emulsion droplet size and its spreadability once they are in contact with the fabric, which is consistent with previous work reported by Nazir and co-wokers.^25^ The PDMS emulsions of high viscosity contain large droplets (hundreds of micron) that would have: (i) strong surface adhesion to the fabric due to the increased contact area; (ii) low penetration profile due to it large size; and (iii) low flowability, which would form a localised deposition profile. As a contrast, PDMS emulsions of low viscosity resulted in droplets of low adhesion to the fabric upon deposition, capable of penetrating through the yarns, that are able to migrate with less resistance, and hence generate a more homogeneous deposition on the target fabric.

**Fig. 4:**
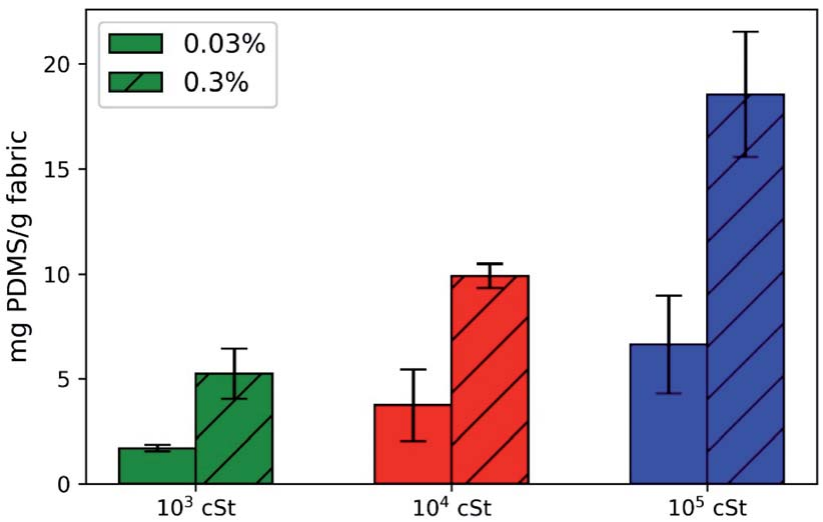
Gravimetric measurements of deposition quantity (PDMS per fabric) as a function of formulation and PDMS viscosity.

Subsequent gravimetric measurements suggest that 0.03% provides a better deposition efficacy than that of 0.3%, despite that the absolute deposition quantity is less, as presented in Fig. 4. There is a noticeable increase of amount deposited as the droplet size increases. The ratio between deposition mass is approximately 1 : 2 : 4 between emulsions prepared by 10^3^, 10^4^, and 10^5^ cSt silicone. Although the collision probability for large droplets is reduced, as rationalised earlier, the volume of each droplet is much greater (*V* ∼ *d*^3^), which compensates the deposition efficacy. Taking into account both collision probability and droplet size, the total volume of 20 μm droplets in the emulsion would be ten times greater than that of 200 μm droplets, and 2.5 times greater than that of 50 μm droplets, assuming that the difference in the density of PDMS is negligible. The discrepancy between our theoretical estimation and the quantities measured experimentally could be attributed primarily to the possibility that the small emulsion droplets can penetrate the gaps between cotton fibres and hence escape back into the bulk solution.

#### SEM-EDX

**Fig. 5:**
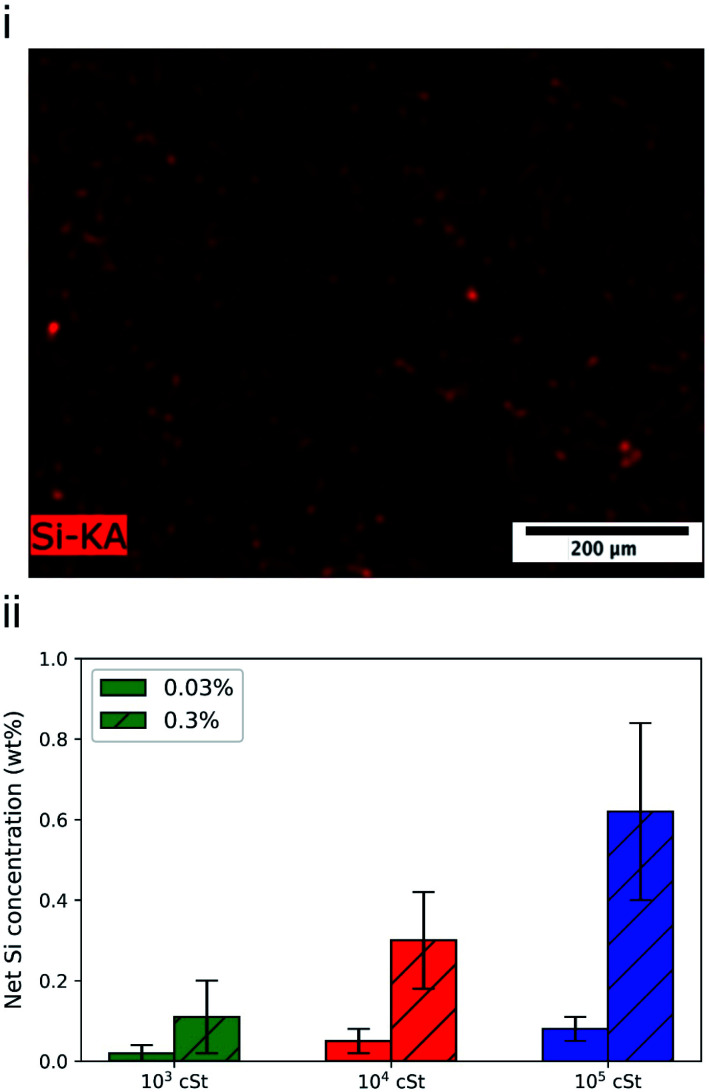
(i) EDX analysis of silicon on the cotton fabric that was exposed to 0.3 wt% PDMS (10^4^ cSt) emulsion, (ii) total amount of silicon signal collected, reporting the presence of PDMS present on the fabric surface, as a function of PDMS formulation used.

The presence of PDMS on fabrics was further evaluated using scanning electron microscopy based elementary analysis. Fig. 5i shows the SEM based element analysis results for a cotton fabric sample treated by a 0.3 wt% 10^3^ cSt PDMS emulsion. Although several primary elements, such as carbon, oxygen, were acquired throughout the measurements, only silicon is shown as its presence confirms the presence and distribution of PDMS across the fabric samples.^56–58^ Signal of silicon on the fabric surface appears to be weak, in comparison to the positive control (data not shown), which is consistent with the fluorescence micrographs.

The deposition profile of PDMS seems to be homogeneous with Si being uniformly detected throughout the fabric surface (Fig. 5i). Similar micrographs were obtained for fabrics treated by the other PMDS emulsions prepared, followed by EDX analysis to quantify the deposited amount of Si (Fig. 5ii). The highest quantities of Si and thus PDMS deposited were detected for the emulsions with the 10^5^ cSt PDMS variant (Fig. 5iii). The higher deposited quantities can be connected to the larger PDMS droplets formed and deposited on the fabric. A large portion of small PDMS droplets will be filtered through the fabric yarns resulting in less PDMS being deposited on or between the fibres. It is striking to note that the ratio between signal intensity is 1 : 2 : 4, which is consistent with the gravimetric data (Fig. 4).

#### Penetration profile of PDMS

Upon contact during the deposition process, PDMS droplets of various size would migrate into the fabrics. At the macroscopic scale when an infinite amount of liquid migrates through the porous fabric, this is commonly known as wicking.^59^ For droplets of confined volume, wicking can be determined by a synergistic effect of Weber number and Reynolds number of the liquid droplet, as discussed earlier. To evaluate such process, contact angle measurements of PDMS on wet cotton fabrics were carried out.

**Fig. 6:**
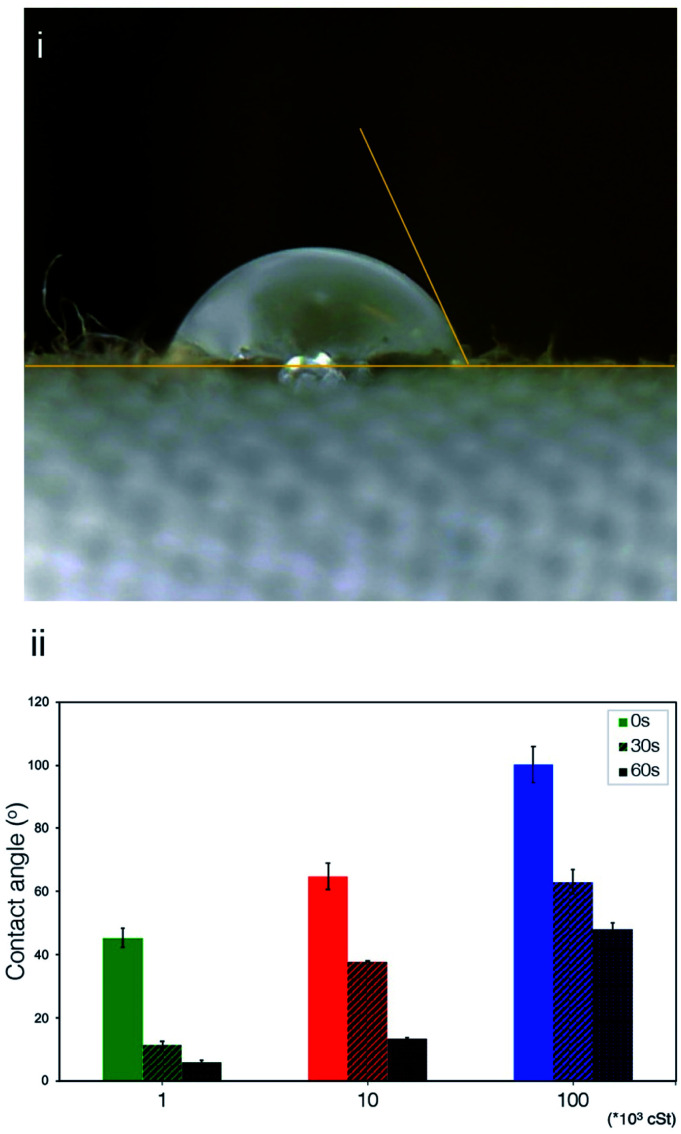
(i) contact angle image of a drop of PDMS placed on the cotton fabric; (ii) contact angle values acquired at 0, 30, and 60s when PDMS drops of various viscosity was placed on a wet cotton fabric.

Fig. 6 shows the contact angle measurements on wet cotton fabric for all PDMS variants (10^3^, 10^4^, and 10^5^ cSt), which were acquired instantaneously, and subsequently at 30 and 60 s upon deposition. PDMS sample of high viscosity (10^5^ cSt) exhibited the greatest initial contact angle, followed by the one with medium viscosity (10^4^ cSt), and the one with low viscosity (10^3^ cSt) presents the lowest contact angle, which confirms the role of viscosity. As summarised by Nyoni and Brook,^59^ the distance of liquid travelled in a fabric is described by6
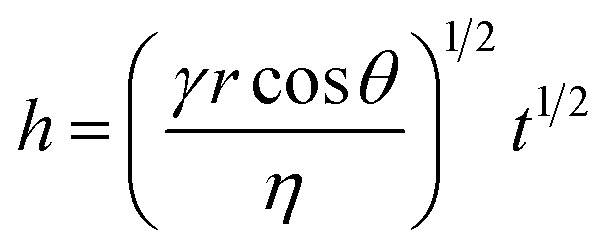
where *γ* is liquid surface tension, *η* is liquid viscosity, *r* is capillary radius, and *θ* is contact angle of the liquid against the fibre substance. Between the PDMS raw materials used in the present work in preparing the emulsion, variation in their density is insignificant (0.930–0.968 g cm^−3^), surface tension is consistent (approximately 21.3 ± 0.2 and 41.4 ± 0.2 mN m^−1^ against air and water, respectively), and hence the viscosity plays the vital role.^55^ This provides a qualitative guidance on the relationship between the timeframe of which the liquid penetrates into the fabric and its viscosity: the PDMS of 10^3^ cSt would penetrate 3.2 times greater than that of 10^4^ cSt and 10 times greater than that of 10^5^ cSt under the same timeframe. It is worth noting that such difference is time dependent. Overall, data presented in Fig. 6ii suggests that droplets of 10^3^ cSt would penetrate much faster into the fabrics than those of 10^4^ or 10^5^ cSt, which supports our hypothesis and the results acquired by both fluorescence microscopy and SEM-EDX.

In recent studies,^60–63^ robust coating based on PDMS or other compounds was developed on textiles to render a superhydrophobic or superamphiphobic characteristics, which was evaluated by using water contact angle (WCA). These studies highlight the importance of controlling surface wettability of textile, which underpins the applications such as oil-water separation and anti-fouling. Unlike the chemical treatment routes discussed, physical deposition of emulsified PDMS was investigated in the present work. However, it is controlled by the same principles discussed early. We would, however, like to highlight that the viscosity of PDMS plays a critical role in such physical deposition process as the surface energy of all three PDMS used in the present work is the same. Another important difference to the previous work^60–63^ where permanent coating was developed on textiles such as cotton fabrics, PDMS layer studied in the present work is not chemically bond to the textile due to the safety regulatory requirements, and hence could be removed in the subsequent washing cycles, although there might be some build-up over the course of a §multiple washing-deposition cycle.

### Mechanical properties and consumer evaluation

**Fig. 7:**
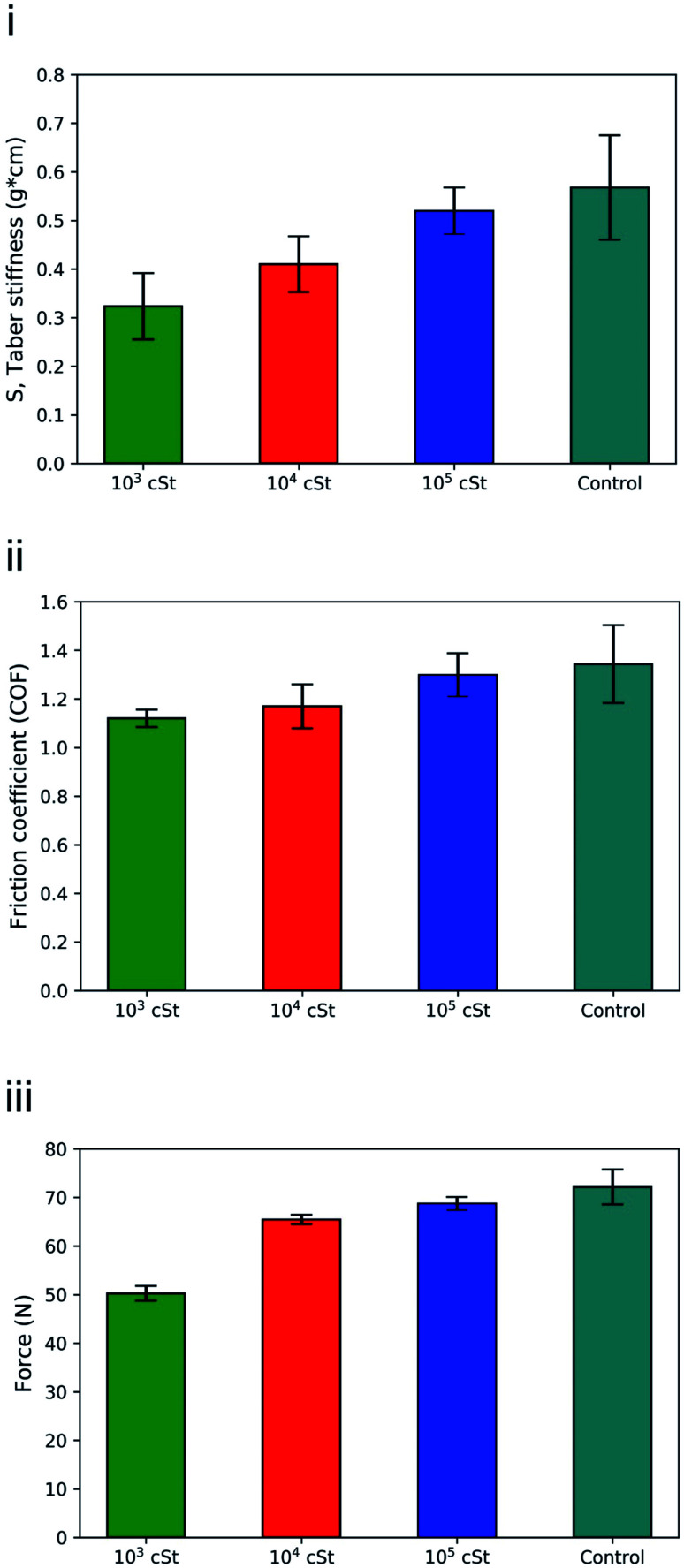
(i) stiffness; (ii) friction, and (iii) compression resistance of cotton fabrics treated by emulsion containing 1, 10, 100 kcSt PDMS.

To assess quantitatively the effect of silicone deposition on the sensorial benefits, a range of mechanical measurements and consumer evaluation were carried out. Fig. 7 shows the normalised values of stiffness, compression and friction measurements of fabrics treated with PDMS emulsions with different PDMS variants (10^3^, 10^4^, and 10^5^ cSt). Overall, it appears that the fabrics treated with PDMS emulsions exhibited better bending properties and lower stiffness values in comparison to the control sample, fabrics washed without PDMS (Fig. 7i). Samples treated with low viscosity PDMS (10^3^ cSt) emulsions exhibited the best bending properties with 44% decrease in stiffness, while those treated with 10^4^ cSt PDMS and 10^5^ cSt PDMS emulsions exhibited 28% and 9% decrease in stiffness respectively. This is likely attributed to the great penetration capability of the low viscosity PDMS into cotton fabric. On the contrary, high viscosity PDMS tends to accumulate on the fabric surface and is not able to provide the same magnitude of bending benefits.

Likewise, it appears that the fabrics treated with the PDMS emulsions required less force to be compressed in comparison to the control (Fig. 7ii), which confirms the effect of PDMS in softening the fabrics. Terry cotton washed with the 10^3^ cSt PDMS emulsion exhibited the highest compressibility, requiring 30% less force (72.16 N *vs.* 50.26 N) as opposed to the fabrics treated with 10^4^ cSt PDMS and 10^5^ cSt PDMS emulsions that required 9% and 5% less force. Both bending and compression are being viewed as critical parameters for consumer tactile benefits. Similar to the wrinkle recovery ability, for which energy dissipation plays a dominant role,^64^ having a thorough coverage of PDMS across the fabric appears to be very important for delivering desired performance of bending and compression properties. Surface analysis carried out by Xu *et al.*,^65^ and colleagues showed silicone adsorbed to a cellulose surface would form a smooth film that can deliver a lubrication function, which significantly reduces the energy required in bending and compressing the fabrics.

Treatment with PDMS emulsions also improved the friction properties of the terry fabrics with a reduced Coefficient of Friction (COF) after treatment (Fig. 7iii). Fabrics washed with emulsions prepared with the low viscosity PDMS (10^3^ cSt) exhibited the lowest friction coefficient values which was 17% lower than that of the control fabrics. Fabrics treated with 10^4^ cSt and 10^5^ cSt PDMS emulsions exhibited a 13% and 3% decrease respectively. From the tribological perspective, our friction measurements involve a low normal load (6.86 N), slow sliding velocity (0.8 cm s^−1^), and small amount of deposited PDMS on the fabric surface, which imply that the friction takes place in the boundary lubrication regime whereby interfacial friction is determined by the interaction between the surfaces in contact.^13,66^ Although silicone of high viscosity could help to reduce interfacial friction, as previous study suggests,^67^ it was carried out in a scenario in which the contact interface was saturated by the silicone oil, which is a distinctively different contact mechanics to what is being discussed in the present work. Such correlation between viscosity and COF would probably be evident if the deposition profile exhibited the same uniformity for all the samples, which contradicts to our experimental observation. It is very likely that the emulsions contained large droplets of PDMS (using high viscosity PDMS) were distributed unevenly on the fabric surface, thus the total decrease in the COF value was not significant. The presence of smaller PDMS droplets in the 10^3^ cSt PDMS emulsions, as evidenced by the size measurements and surface characterisation, resulted in a more uniform distribution on the fabric surface is linked to the decrease on the COF values, which agrees with findings reported previously.^28^ The result supports our explanation that smaller particles can easily penetrate into the fibres and provide coverage and thus less friction between fibres and yarns.

#### Sensorial feel measurements

**Fig. 8:**
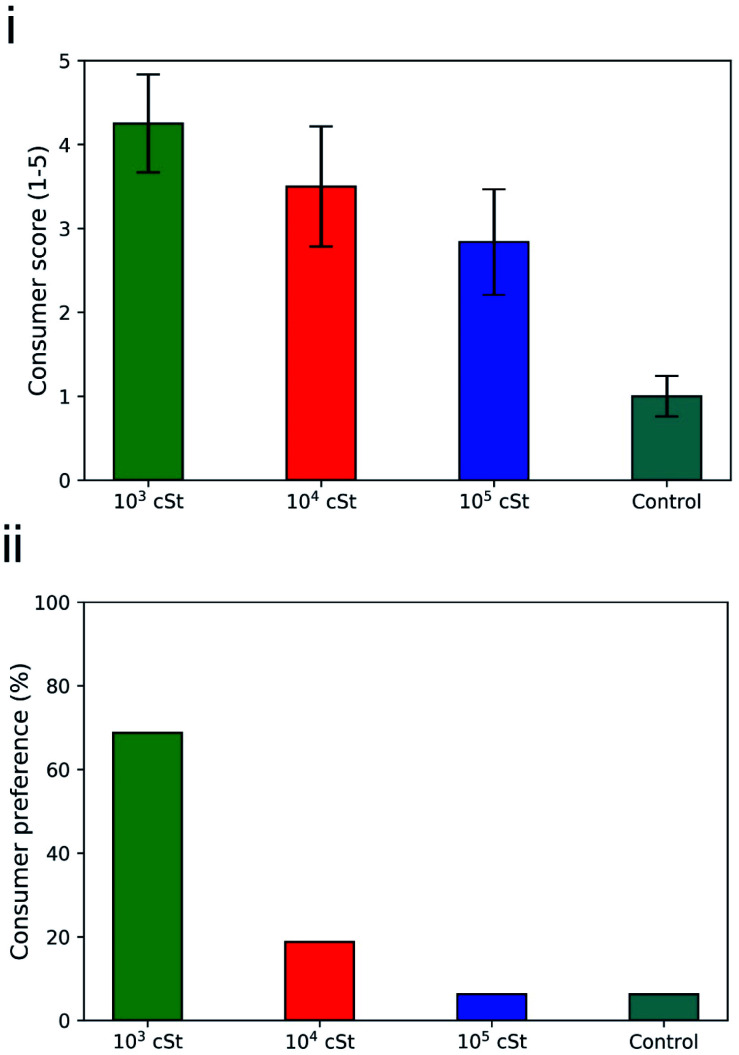
(i) Consumer grading and (ii) consumer preference on the fabrics treated by the PDMS emulsions prepared in the present work, based on an evaluation of 16 adults.

Previous studies^68^ confirm that there is correlation between fabric friction and subjectively perceived touch properties, in particularly for knitted fabrics, whilst other properties such as bending, thickness, and compressibility also contribute. A panel of 16 individuals was asked to evaluate the softness and feel of PDMS treated fabrics. Consumer grading and preference results are presented in Fig. 8, with consumer softness scores presented in Fig. 8i, and consumer preference in Fig. 8ii.

Fabrics washed with 10^3^ cSt PDMS emulsions obtained an average score of 4.25 ± 0.58 from the consumer panel with 5 being the highest score and 1 the lowest possible score. Fabrics treated with 10^4^ cSt and 10^5^ cSt PDMS emulsions received 3.5 ± 0.72 and 2.84 ± 0.63 respectively. The control terry fabric that did not undergo any treatment received the lowest score from the panellists 1.12 ± 0.24. Such result confirms that the surface deposited PDMS can be perceived in terms of tactile sensorial benefits, and that the PDMS of 10^3^ cSt is the most favourable one, followed by 10^4^ cSt and then 10^5^ cSt. Overall consumer preference was also acquired, where the consumers were asked to choose only one of the treated fabric samples based on overall feel (Fig. 8ii). It was found that 69% (11/16) of panellists preferred the feel fabrics washed with 1 kcSt PDMS solutions, 19% (3/16) showed preference for the fabrics washed with 10 kcSt, and 6% (1/16) for the fabrics washed with 10^5^ cSt and 6.25% (1/16). The majority of consumers preferred the fabrics washed with 10^3^ cSt emulsions which are the samples that exhibited the lowest stiffness, lowest compression force and lowest friction coefficient values.

The results from both consumer grading and consumer preference are consistent with the results produced by the mechanical testing, which highlights, once again, the importance of not only deposition profile on the exterior surface of the fabrics but also the penetration characteristics of the oil component into the fabrics. And it is safe to conclude that controlling droplet size by changing the viscosity of a material or altering the formulation process of a product can improve the on-fabric deposition and retention improving the surface properties and leading to higher consumer acceptance.

## Conclusions

In this work, we demonstrated that a coherent design framework that links formulation preparation of model silicone emulsion, their deposition profile on cotton fabrics, the resulting mechanical properties, and corresponding consumer evaluation.

First of all, there is an explicit correlation between emulsion properties and PDMS deposition profile. Properties such as droplet size, viscosity, and concentration have a synergistic and significant impact on the deposition profile of PDMS on fabric. PDMS of medium and high viscosity (10^4^ cSt, 10^5^ cSt) lead to the formation of large PDMS droplets (100–500 μm) in the emulsion, which resulted in an increased PDMS deposition on the fabric surface. However, such large PDMS droplets with high viscosity resulted in a localised, non-even deposition profile on the fabric surface. As a contrast, emulsion prepared using PDMS of low viscosity (10^3^ cSt) contains small droplets that were able to deposit and penetrate the fabrics, despite that the total amount of deposition is less than the counterparts of high viscosity. The subsequent mechanical properties measurements and sensorial feel evaluation confirm the superiority of low viscosity PDMS as a raw material during emulsion preparation *versus* the use of PDMS with higher viscosity. Formulation using 10^3^ cSt PDMS emulsion improved bending, compression and friction properties of the cotton fabric, which was well perceived by the consumer.

To conclude, we would recommend a holistic approach for designing formulated products that contains silicone, or alternative oils, in the future by considering the synergistic effect of oil viscosity, resulting droplet size, interfacial energy on the fibre in contact under the relevant environment, in addition to the other parameters such as emulsifiers used, to accomplish the maximum consumer benefits with minimal raw materials used.

## Conflicts of interest

The authors hereby confirm that there is no conflict to declare.

## Supplementary Material
